# Effects of Thrombomodulin in Reducing Lethality and Suppressing Neutrophil Extracellular Trap Formation in the Lungs and Liver in a Lipopolysaccharide-Induced Murine Septic Shock Model

**DOI:** 10.3390/ijms22094933

**Published:** 2021-05-06

**Authors:** Yu Kato, Osamu Nishida, Naohide Kuriyama, Tomoyuki Nakamura, Takahiro Kawaji, Takanori Onouchi, Daisuke Hasegawa, Yasuyo Shimomura

**Affiliations:** 1 Department of Anesthesiology and Critical Care Medicine, Fujita Health University School of Medicine, 1-98 Dengakugakubo, Kutsukake-cho, Toyoake 470-1192, Japan; accm@fujita-hu.ac.jp (Y.K.); nishida@fujita-hu.ac.jp (O.N.); kuriyama@fujita-hu.ac.jp (N.K.); tomo-n@fujita-hu.ac.jp (T.N.); medcompass@gmail.com (T.K.); hasegawa.daisuke.0407@gmail.com (D.H.); 2 Center for Joint Research Facilities Support, Research Promotion and Support Headquarters, Fujita Health University, 1-98 Dengakugakubo, Kutsukake-cho, Toyoake 470-1192, Japan; tonouchi@fujita-hu.ac.jp

**Keywords:** neutrophil extracellular traps (NETs), sepsis, thrombomodulin, lipopolysaccharide (LPS), organ dysfunction

## Abstract

Neutrophil extracellular trap (NET) formation, an innate immune system response, is associated with thrombogenesis and vascular endothelial injury. Circulatory disorders due to microvascular thrombogenesis are one of the principal causes of organ damage. NET formation in organs contributes to the exacerbation of sepsis, which is defined as a life-threatening organ dysfunction caused by a dysregulated host response to infection. We have previously reported that recombinant human soluble thrombomodulin (rTM) reduces lipopolysaccharide (LPS)-induced NET formation in vitro. Here, we aimed to show that thrombomodulin (TM)-mediated suppression of NET formation protects against organ damage in sepsis. Mice were injected intraperitoneally (i.p.) with 10 mg/kg LPS. rTM (6 mg/kg/day) or saline was administered i.p. 1 h after LPS injection. In the LPS-induced murine septic shock model, extracellular histones, which are components of NETs, were observed in the liver and lungs. In addition, the serum cytokine (interleukin-1β (IL-1β), interleukin-6 (IL-6), tumor necrosis factor-α (TNF-α), macrophage chemotactic protein-1 (MCP-1), and interleukin-10 (IL-10)) levels were increased. The administration of rTM in this model prevented NET formation in the organs and suppressed the increase in the levels of all cytokines except IL-1β. Furthermore, the survival rate improved. We provide a novel role of TM in treating inflammation and NETs in organs during sepsis.

## 1. Introduction

Recent studies have reported the formation of neutrophil extracellular traps (NETs) by neutrophils, which presents a new type of innate immune response [1]. NETs are reticular structures composed of various intracellular neutrophil components such as histones and nuclear DNA, and cytoplasmic antibacterial proteins such as elastase and myeloperoxidase. These structures are released by neutrophils to capture and kill cells [1]. NET formation plays an important role in confining infections to small areas via its involvement in coagulation and thrombogenesis. Hence, the role of NET formation in immunothrombosis has attracted attention [2].

On the other hand, NET formation induces vascular endothelial injury and microvascular circulation disorders [3,4]. In rapidly progressing systemic inflammation, such as sepsis, this immune response is induced not only in the local infection region, but also in the non-infection region, and develops into organ failure.

Thrombomodulin (TM), a multiple domain membrane protein primarily expressed in vascular endothelial cells [5], has been shown to possess anticoagulant [6], anti-inflammatory, and vascular-endothelial-protective activities [7,8]. Because the expression of TM is reduced at the onset of septic disseminated intravascular coagulation (DIC) [9], organ dysfunction during sepsis could be associated with NET formation in various organs.

Sepsis is defined as life-threatening organ dysfunction caused by a dysregulated host response to infection [10], is a major cause of mortality worldwide, and urgently requires the establishment of effective treatment methods. The mortality rate is extremely high when sepsis is complicated by DIC [11,12,13].

Using an in vitro study involving human neutrophil and platelet cultures, we have reported that recombinant human soluble thrombomodulin (rTM) suppresses lipopolysaccharide (LPS)-induced NET formation [14], and we have hypothesized that by suppressing NET formation, TM prevents organ damage during sepsis onset, thereby improving survival rates. To verify this hypothesis, the effects of TM on the survival rate, NET formation in the lungs and liver, and serum cytokine concentration were evaluated using a LPS-induced murine septic shock model.

## 2. Results

### 2.1. Effects of rTM Administration on Survival Rate in the LPS-Induced Murine Septic Shock Model

**Figure 1:**
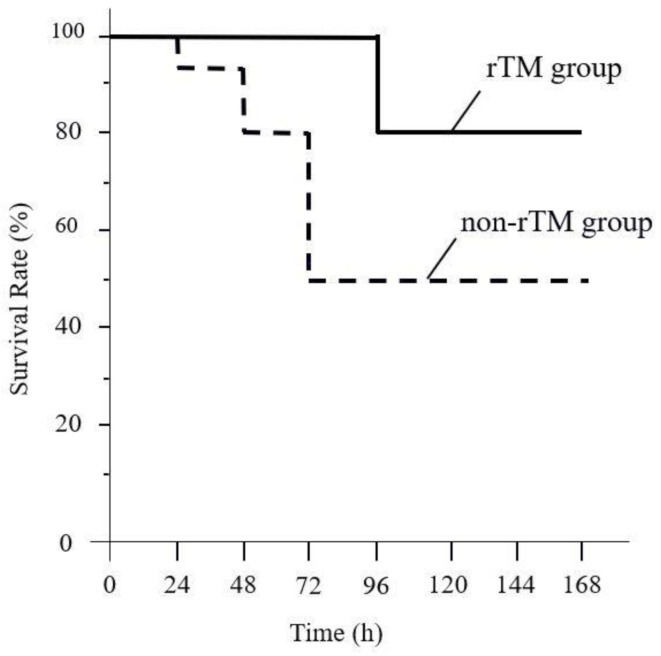
Effect of recombinant human soluble thrombomodulin (rTM) on survival in a lipopolysaccharide (LPS)-induced murine septic shock model. LPS (10 mg/kg) is injected into the peritoneal cavity (non-rTM group); rTM (6 mg/kg/day) is administered 1 h after the LPS injection (rTM group). The survival of non-rTM (*n* = 20) and rTM groups (*n* = 10) is monitored for up to 7 days. Values are compared between the non-rTM group and the rTM group. *p* < 0.05.

The survival rates of mice in each treatment group after 72 h are shown in Figure 1. The mice in the non-rTM group began to die 24 h after LPS administration, and the mortality rate reached 50% after 72 h, whereas all mice in the rTM group survived 72 h after rTM administration.

### 2.2. Effects of rTM Administration on NET Formation in Organs in the LPS-Induced Murine Septic Shock Model

**Figure 2:**
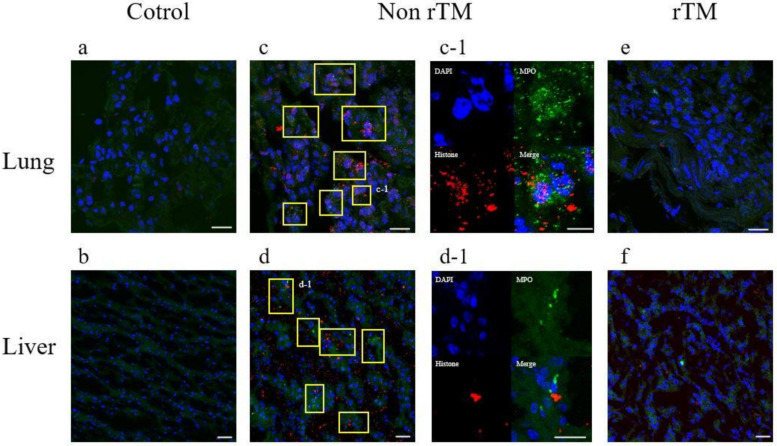
Suppressive effect of rTM on lung and liver neutrophil extracellular trap (NET) formation. LPS (10 mg/kg) is injected into the peritoneal cavity (non-rTM group); rTM (6 mg/kg/day) is administered 1 h after the LPS injection (rTM group). As a control, physiological saline is administered instead of LPS and rTM (control group). The lung and liver tissues are collected from mice 8 h after the administration of LPS or saline. Immunofluorescence staining for myeloperoxidase (MPO) and histone H2A.X is performed, and the nuclei are stained with 4′,6-diamidino-2-phenylindole (DAPI): (**a**) control lung, (**b**) control liver, (**c**) non-rTM lung, (**c-1**) enlarged image of non-rTM lung, (**d**) non-rTM liver, (**d-1**) enlarged image of non-rTM liver, (**e**) rTM lung, and (**f**) rTM liver. Blue—DAPI; green—MPO; red—histone H2A.X. NETs—yellow square. Magnification: ×630 (lung), ×200 (liver); scale bar: 20 μm (**a**,**c**,**e**), 10 μm (**c-1**), 40 μm (b,d,f), 20 μm (**d-1**).

NET formation was observed using immunofluorescence staining of tissue samples collected 8 h after rTM administration to the LPS-induced murine septic shock model. To confirm the formation of NETs within the lung and liver, we performed immunofluorescence staining of the left lung section and the left liver lateral lobe, respectively. In the control group, no NET formation was observed in the lung and liver samples (Figure 2a,b). In the non-rTM group, histones and myeloperoxidase (MPO) were present and NETs were formed in the lungs and liver (Figure 2c,d). NETs were identified by neutrophil components co-localized with MPO (green) and extracellular histones (red). NET is indicated by a yellow square. In the rTM group, the levels of histones and MPO were lower than that in the non-rTM group (Figure 2e,f), confirming that NET formation was suppressed.

### 2.3. Assessment of NET Morphology in the Liver of LPS-Induced Murine Septic Shock Model

**Figure 3:**
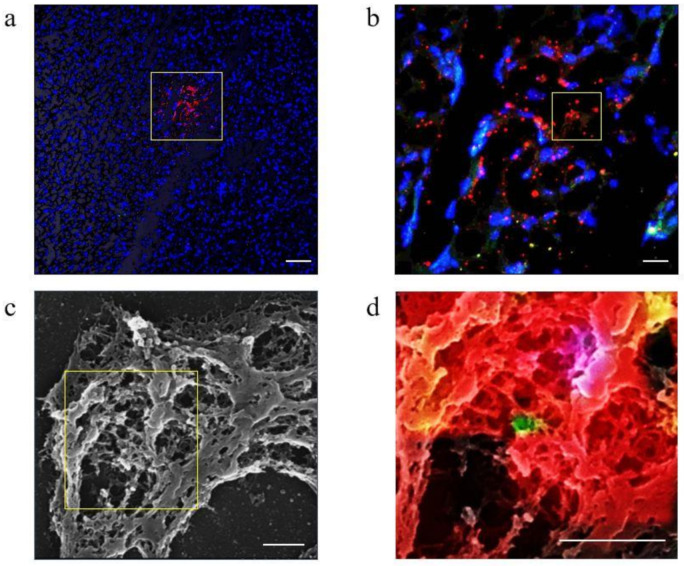
Confirmation of NETs formation in the liver using confocal microscopy and SEM images. Saline is administered to LPS-induced septic shock model mice 1 h after the LPS administration, and the livers are observed after 8 h using confocal microscopy and Scanning Electron Microscope (SEM). Immunofluorescence staining for MPO (green) and histone H2A.X (red) are performed, and nuclei are stained with DAPI (blue); the three colored images are merged. (**a**) Magnification: ×100; scale bar = 20 μm; (**b**) magnification: ×400; scale bar = 80 μm. Sections are sputter-coated with gold palladium and observed using SEM after confocal microscopy. (**c**) Magnification: ×7000; scale bar = 2 μm. (**d**) The SEM and confocal images are merged). Magnification: ×7000; scale bar = 250 nm. The yellow box indicates enlargement.

A physiological saline solution was administered to the LPS-induced murine septic shock model, and hepatic tissue samples were collected after 8 h. NET formation in the liver was confirmed using immunofluorescence staining (Figure 3a,b). To observe the morphology of the NET reticular structure, the same sites that had been imaged after immunofluorescence staining were studied using Scanning Electron Microscope (SEM) (Figure 3c). The images from both the confocal fluorescence microscopy and SEM were merged, enabling confirmation of the presence of histone and MPO granules in the three-dimensional NET reticular structures (Figure 3d).

### 2.4. Estimation of Blood Cytokine Levels in the LPS-Induced Murine Septic Shock Model

**Figure 4:**
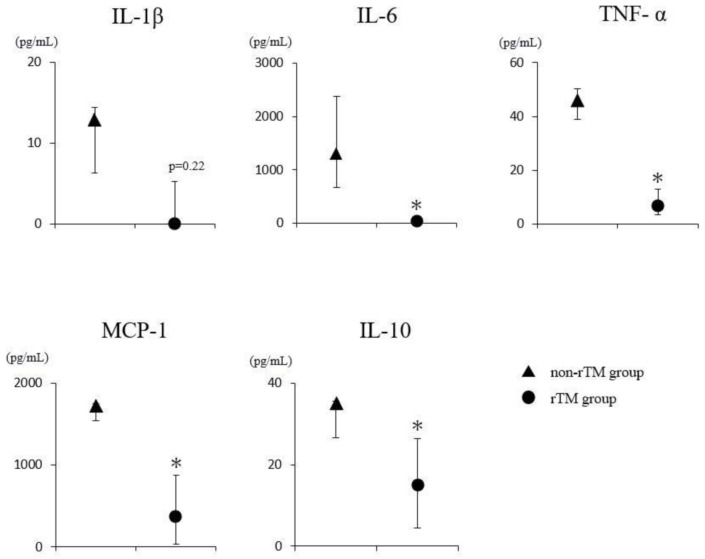
Effects of rTM on the serum cytokine levels. LPS (10 mg/kg) is injected into the peritoneal cavity (non-rTM group); rTM (6 mg/kg/day) is administered 1 h after the LPS injection (rTM group). Blood is collected from both groups 36 h after the LPS administration and the serum is prepared. IL-1β, IL-6, TNF-α, MCP-1, and IL-10 levels are determined using cytometric bead array (CBA). Values are expressed as median ± interquartile range (IQR), and compared between the non-rTM and rTM groups using Mann–Whitney U test. * *p* < 0.05 ▲ = non-rTM group (*n* = 5), ● = rTM group (*n* = 5).

Changes in the serum cytokine levels 36 h after the administration of rTM to the LPS-induced murine septic shock model were determined using cytometric bead array (CBA). The results in the non-rTM group were as follows: Interleukin (IL)-1β: 12.92 pg/mL; IL-6: 1307.88 pg/mL; tumor necrosis factor (TNF)-α: 45.99 pg/mL; macrophage chemotactic protein (MCP-1): 1634.53 pg/mL; and IL-10: 34.98 pg/mL. The results in the rTM group were as follows: IL-1β: 0.00 pg/mL; IL-6: 25.30 pg/mL; TNF-α: 6.70 pg/mL; MCP-1: 365.50 pg/mL; and IL-10: 15.05 pg/mL. Thus, the blood levels of IL-1β, IL-6, TNF-α, MCP-1, and IL-10 increased as a result of LPS administration. The administration of rTM in the LPS-induced murine septic shock model suppressed the increase in the levels of all cytokines, except IL-1β. (Figure 4).

## 3. Discussion

In this study, we observed that administration of rTM improved the survival rate of LPS-induced mice with sepsis. In this model, NETs were formed in the lungs and liver, and the serum levels of cytokines IL-1β, TNFα, IL-6, MCP-1, and IL-10 increased. Administration of rTM suppressed NET formation in the lungs and liver, and suppressed the increase in the levels of all cytokines, except IL-1β.

TM is a single-pass membrane protein with 575 amino acids harboring five domains (D1–5). D2 or the EGF-like domain contains epidermal growth factor-like repeats and six regions, numbered EGF1 to EGF6. Studies have shown that TM possesses anticoagulant, anti-inflammatory, and vascular-endothelial-protective activities. The mechanism underlying TM action is believed to be the following: thrombin, which induces coagulation and inflammation, binds to EGF5 and EGF6 of TM, forming a thrombin–thrombomodulin complex, resulting in the inactivation of thrombin [15]. In addition, the thrombin–thrombomodulin complex activates protein C, thereby inducing anti-inflammatory and vascular-endothelial-protective effects; these effects are mediated by endothelial cell protein C and protease-activated receptor-1 [13,16]. D1, the lectin-like domain of TM, binds to and neutralizes high-mobility group box 1 (HMGB1) and histones, acting as typical damage-associated molecular patterns (DAMPs), as well as to LPS, as typical pathogen-associated molecular patterns (PAMPs), thereby preventing thrombosis and inflammation [17,18,19].

NET formation is an innate immune response aimed at preventing pathogen dispersion; however, it may cause microvascular obstruction and induce tissue damage [20]. In cases of continued tissue damage, DAMPs (histones, HMGB1, etc.) released by tissues may induce the formation of new NETs and cause organ damage.

Extracellular histones activate platelets and promote coagulation, confining infection to one area. In addition, extracellular histones exert their antimicrobial effects in a manner similar to other antimicrobial peptides [21]. However, it has been reported that extracellular histones released in response to inflammatory challenge contribute to endothelial dysfunction, organ failure, and death during sepsis [22,23,24].

The intravenous administration of histones in mice markedly increases neutrophil accumulation, hemorrhage, and thrombogenesis in the lungs. However, when anti-histone antibodies are administered, this damage is ameliorated [25]. Other studies showed that rTM administration in mice treated with histones resulted in the suppression of intravascular thrombogenesis and alleviation of the decrease in platelet numbers, thereby rescuing the mice from life-threatening thrombosis [18].

In the present study, histones were found to be dispersed throughout the lungs and liver in the LPS-induced murine septic shock model, and the presence of NETs in the liver was confirmed using high-magnification microscopy. However, no histones were detected in the rTM group, suggesting that TM administration suppresses histone-induced organ damage, and may thus contribute to an improvement in the survival rate.

The lungs and liver are the target organs of sepsis, and neutrophil accumulation has been confirmed in these damaged organs [26]. In neutrophil-deficient mice, platelet accumulation is not observed in the lungs or liver [27]. Neutrophils bind to platelets, resulting in the release of NETs [28]; hence, NETs are possibly involved in these types of organ damage.

The NETs formation caused by activated platelets is involved in the systemic immunothrombosis, microvascular thrombosis in organs, and endothelial dysfunction in sever sepsis. Increased thrombin during sever sepsis plays an essential role in activating platelets. Thrombomodulin binds to thrombin to form a complex that inactivates the coagulant activity of thrombin and activates protein C [15]. Therefore, TM could inhibit activation of platelets and NET formation by inactivating thrombin. Moreover, TM bind to and inactivate HMGB1 [17] and Histones [18]. The present findings suggest that rTM can inhibit LPS-induced NET formation in organs by binding with thrombin, HMGB1 and Histones.

In vivo, blood passes through the lungs, which, owing to their role in gaseous exchange, are in direct contact with the external environment. The liver’s contact with the external environment is mediated by the gastrointestinal tract. Approximately 60% of the bacteria in the bloodstream are captured in the platelet-neutrophil accumulation sites in the lungs and liver [26,28], after which the blood returns to the rest of the body; thus, the lungs and liver function as filters for removing pathogens from the body. As the lungs and liver are involved in the functioning of the immune system and are rich in low-pressure microcirculation, bacteria are readily captured as a result of NET formation. However, the anomalous induction of NET formation in the organs leads to organ damage; therefore, TM plays an important role in the alleviation of organ damage, which is closely linked to the prognosis of sepsis.

Previous studies have reported NET formation in the lungs of mice with acute respiratory distress, prepared by spraying hydrochloric acid into the bronchi [29], and in the damaged liver of mice after ischemic reperfusion [30]. Sepsis involves the progression of organ damage, including organs remote from the primary infection focus, which contributes to its severity and leads to multiple organ failure. An evaluation of the organs far away from the infection site in mice intraperitoneally administered LPS will be useful for elucidating the pathology of sepsis and developing appropriate treatment methods.

In the LPS-induced murine septic shock model used in this study, the levels of IL-6, TNFα, MCP-1, and IL-10 increased 36 h after LPS administration, except IL-1β. The levels of IL-1β in non-rTM group was too low to evaluate the inhibitory effect of rTM. However, rTM administration suppressed the increase in these cytokine levels. The administration of rTM 8 h after LPS administration suppressed NET formation in the lungs and liver. Possibly, the inhibition of NET formation within organs prevented the spread of immunothrombosis, tissue damage, and microvascular obstruction, resulting in the suppression of inflammation and an improvement in the mouse survival rate.

The LPS-induced murine septic shock model was prepared using LPS, which is a type of PAMP. LPS administration has been used previously for preparing septic shock models; however, the pathological state of the sepsis in this model differs from that in actual patients [31], and a sepsis and sepsis-related organ damage model was therefore put forward [32]. In addition, whether the effects of rTM observed in this study are identical to those of the TM formed in the body is not known. These issues, therefore, remain to be investigated.

## 4. Materials and Methods

### 4.1. Animals and Study Design

Six- to eight-week-old female C57BL/6J Jms mice were purchased from SLC, Inc. (Hamamatsu, Japan). LPS (Escherichia coli 0111, 125-05201) was purchased from Wako Pure Chemical Industries Ltd. (Osaka, Japan). rTM (ART-123) was used for the experimental studies, and was provided by Asahi Kasei Pharma Corp. (Tokyo, Japan). The mice were injected intraperitoneally (i.p.) with 10 mg/kg LPS. rTM (6 mg/kg/day, i.p.; rTM group) or saline (non-rTM group) was injected 1 h after the LPS injection. As a control, an equal volume of physiological saline was administered instead of LPS and rTM (control group). Survival was monitored for up to 7 days. We calculated the number of samples required in survival rates using Cox’s proportional hazards model. After LPS or saline was injected for 8 or 36 h, the mice were anesthetized with isoflurane and were euthanized for the collection of blood, lung, and liver samples. Septic shock mice exhibited clinical sepsis symptoms (lethargy, piloerection, reduced interest in food and water, and hunched posture).

### 4.2. Ethics Statement

The mice were handled ethically according to the Regulations for the Management of Laboratory Animals at Fujita Health University. The experimental protocol for the ethical use of these animals was approved by the Animal Care and Use Committee at Fujita Health University (permit no. AP16088, 6 July 2016). Any mice exhibiting loss of righting reflex when placed in a supine position were identified as moribund and were immediately euthanized.

### 4.3. Immunofluorescence Staining of Lung and Liver

Frozen blocks of murine lung and liver tissue were sectioned to a thickness of 5 μm and mounted on glass slides. After fixation with 4% formaldehyde and blocking, the sections were incubated with anti-myeloperoxidase (MPO; ab45977, Abcam, Cambridge, MA, USA) and histone H2A.X antibodies (sc-54607, Santa Cruz Biotechnology, Santa Cruz, CA, USA) for 1 h at 37 °C. After washing with 0.1% Tween 20, the samples were incubated with Alexa Fluor 488 secondary antibodies (A21206, Thermo Fisher Scientific, Waltham, MA, USA) and Alexa Fluor 555 secondary antibodies (A21432, Thermo Fisher Scientific, Waltham, MA, USA) for 1 h at 37 °C, and were mounted using a ProLong Gold antifade mountant with 4′,6-diamidino-2-phenylindole (DAPI; Life Technologies, Carlsbad, CA, USA) to detect DNA. The samples were observed using a confocal laser scanning microscope (LSM710, Carl Zeiss, Oberkochen, Germany).

### 4.4. Confocal Laser Scanning Microscopy and Scanning Electron Microscopy (SEM) of the Liver

Murine livers were paraffin-embedded and sectioned after being fixed in 4% paraformaldehyde. Paraffin sections of a 3 μm thickness were mounted on glass slides, deparaffinized with xylene, and rehydrated in graded ethanol. The section was incubated with a mixture of anti-MPO (ab45977, Abcam, Cambridge, MA, USA) and histone H2A.X antibodies (sc-54607, Santa Cruz Biotechnology, Santa Cruz, CA, USA ) overnight at 25–26 °C, followed by incubation with a mixture of Alexa Fluor 488 secondary antibodies (A21206, Thermo Fisher Scientific, Waltham, MA, USA), Alexa Fluor 555 secondary antibodies (A21432, Thermo Fisher Scientific, Waltham, MA, USA), and DAPI (D1306, Life Technologies, Carlsbad, CA, USA) for 1 h at 25–26 °C. The section was dehydrated in graded ethanol and tert-butyl alcohol, dried in a freeze-drying apparatus (JFD-310, JEOL, Tokyo, Japan), and observed without using a cover slip on a confocal laser scanning microscope (LSM710, Carl Zeiss, Oberkochen, Germany). The section was then sputter-coated with gold palladium using JFC-1500 (JEOL) and observed on a SEM (S-4000, Hitachi, Tokyo, Japan). Confocal laser scanning microscopic and SEM images were merged using imaging software (Adobe Photoshop CS5.1, Adobe Systems, San Jose, CA, USA).

### 4.5. Measurement of Cytokine Levels

The mice were anesthetized using isoflurane and blood was collected after cardiac puncture. The blood samples were centrifuged at 1400× *g* for 15 min at 4 °C, and stored as serum at 80 °C until use. A cytometric bead array (CBA) mouse inflammation kit (BD Biosciences, San Jose, CA) was used to determine the interleukin-1β (IL-1β), interleukin-6 (IL-6), tumor necrosis factor-α (TNF-α), macrophage chemotactic protein-1 (MCP-1), and interleukin-10 (IL-10) levels in the serum. The assay was performed according to the instructions provided with the kit and the beads were analyzed on a FACS Calibur flow cytometer (BD Biosciences, San Jose, CA, USA).

### 4.6. Statistics

A Kaplan–Meier curve was plotted and the survival difference was examined using the log-rank test. The levels of cytokines were compared between the non-rTM and rTM groups using a Mann–Whitney U test. *p* < 0.05 indicated statistically significant differences. Stat Flex version 5 (Artech Corporation, Osaka, Japan) was used for the statistical analyses.

## 5. Conclusions

These results suggest that rTM played a novel role in NET formation and was effective in the LPS-induced septic shock model by suppressing NET formation in the organs and the level of cytokines in the blood, which resulted in a prevention and decrease in lethality.

## Data Availability

The data presented in this study are available on request from the corresponding author. The data are not publicly available due to intellectual property/confidentiality issues.
